# Supplementary material for: Individuals with Inflammatory Bowel Disease Have an Altered Gut Microbiome Composition of Fungi and Protozoa
Source: Microorganisms. 2022 Sep 26;10(10):1910. doi: 10.3390/microorganisms10101910 (PMC9610947; doi:10.3390/microorganisms10101910)
Supplement: Supplementary file 1 [file microorganisms-10-01910-s001.zip › microorganisms-1894217-supplementary.pdf]

Supplemental Materials

for

Individuals with Inflammatory Bowel Disease Have an Altered Gut Microbiome Composition of Fungi and Protozoa

Gina L. Guzzo, Murthy N. Mittinty, Bastien Llamas, Jane M. Andrews, and Laura S. Weyrich

Table S1. Results of RiboTagger for the 1000IBD and 500FG studies.

Sample ID	Disease status	Confidence	Kingdom	Phylum	Class	Order	Family	Genus	18S V region
1000IBD00010	IBD	1	Eukaryota	Ascomycota	Saccharomycetes	Saccharomycetales	Saccharomycetaceae	Saccharomyces	v4
1000IBD00107	IBD	1	Eukaryota	Ascomycota	Saccharomycetes	Saccharomycetales	Saccharomycetaceae	Saccharomyces	v4
1000IBD00198	IBD	1	Eukaryota					Blastocystis	v4
1000IBD00349	IBD	1	Eukaryota					Blastocystis	v4
1000IBD00401	IBD	1	Eukaryota	Ascomycota	Saccharomycetes	Saccharomycetales	Saccharomycetaceae	Saccharomyces	v4
1000IBD00510	IBD	1	Eukaryota					Blastocystis	v4
1000IBD00541	IBD	1	Eukaryota	Parabasalia	Tritrichomonadea			Dientamoeba	v4
1000IBD00556	IBD	1	Eukaryota	Ascomycota	Saccharomycetes	Saccharomycetales	Saccharomycetaceae	Saccharomyces	v4
1000IBD00613	IBD	1	Eukaryota					Blastocystis	v4
1000IBD00394	IBD	1	Eukaryota	Ascomycota	Saccharomycetes	Saccharomycetales	Dipodascaceae	Galactomyces	v4
1000IBD00476	IBD	1	Eukaryota					Blastocystis	v4
1000IBD00581	IBD	1	Eukaryota	Ascomycota	Saccharomycetes	Saccharomycetales	Saccharomycetaceae	Saccharomyces	v4
1000IBD00675	IBD	0.89	Eukaryota	Ascomycota	Saccharomycetes	Saccharomycetales	Saccharomycetaceae	Debaryomyces	v4
1000IBD01328	IBD	1	Eukaryota	Ascomycota	Saccharomycetes	Saccharomycetales	Saccharomycetaceae	Saccharomyces	v4

1000IBD00010	IBD	0.89	Eukaryota	Ascomycota	Saccharomycetes	Saccharomycetales	Saccharomycetaceae		v5
1000IBD00107	IBD	0.89	Eukaryota	Ascomycota	Saccharomycetes	Saccharomycetales	Saccharomycetaceae		v5
1000IBD00198	IBD	1	Eukaryota					Blastocystis	v5
1000IBD00208	IBD	1	Eukaryota					Blastocystis	v5
1000IBD00349	IBD	1	Eukaryota					Blastocystis	v5
1000IBD00401	IBD	0.89	Eukaryota	Ascomycota	Saccharomycetes	Saccharomycetales	Saccharomycetaceae		v5
1000IBD00431	IBD	1	Eukaryota					Blastocystis	v5
1000IBD00510	IBD	1	Eukaryota					Blastocystis	v5
1000IBD00556	IBD	0.89	Eukaryota	Ascomycota	Saccharomycetes	Saccharomycetales	Saccharomycetaceae		v5
1000IBD00569	IBD	0.89	Eukaryota	Ascomycota	Saccharomycetes	Saccharomycetales	Saccharomycetaceae		v5
1000IBD00613	IBD	1	Eukaryota					Blastocystis	v5
1000IBD00476	IBD	1	Eukaryota					Blastocystis	v5
1000IBD00581	IBD	0.89	Eukaryota	Ascomycota	Saccharomycetes	Saccharomycetales	Saccharomycetaceae		v5
1000IBD00675	IBD	1	Eukaryota	Ascomycota	Saccharomycetes	Saccharomycetales			v5
1000IBD00198	IBD	1	Eukaryota					Blastocystis	v6
1000IBD00349	IBD	1	Eukaryota					Blastocystis	v6

1000IBD00431	IBD	1	Eukaryota						v6
1000IBD00492	IBD	1	Eukaryota	Parabasalia	Tritrichomonadea			Dientamoeba	v6
1000IBD00510	IBD	1	Eukaryota						v6
1000IBD00556	IBD	0.99	Eukaryota	Ascomycota					v6
1000IBD00610	IBD	1	Eukaryota	Metazoa					v6
1000IBD00613	IBD	1	Eukaryota						v6
1000IBD00476	IBD	1	Eukaryota					Blastocystis	v6
1000IBD00581	IBD	0.99	Eukaryota	Ascomycota					v6
1000IBD00662	IBD	1	Eukaryota	Parabasalia	Tritrichomonadea			Dientamoeba	v6
1000IBD01329	IBD	1	Eukaryota					Blastocystis	v6
1000IBD00006	IBD	1	Eukaryota	Metazoa					v7
1000IBD00010	IBD	0.85	Eukaryota	Ascomycota	Saccharomycetes	Saccharomycetales	Saccharomycetaceae	Saccharomyces	v7
1000IBD00035	IBD	1	Eukaryota	Ascomycota	Saccharomycetes	Saccharomycetales	Dipodascaceae	Galactomyces	v7
1000IBD00107	IBD	0.85	Eukaryota	Ascomycota	Saccharomycetes	Saccharomycetales	Saccharomycetaceae	Saccharomyces	v7
1000IBD00198	IBD	1	Eukaryota					Blastocystis	v7
1000IBD00206	IBD	1	Eukaryota					Blastocystis	v7

1000IBD00349	IBD	1	Eukaryota					Blastocystis	v7
1000IBD00401	IBD	0.85	Eukaryota	Ascomycota	Saccharomycetes	Saccharomycetales	Saccharomycetaceae	Saccharomyces	v7
1000IBD00431	IBD	1	Eukaryota					Blastocystis	v7
1000IBD00510	IBD	1	Eukaryota					Blastocystis	v7
1000IBD00556	IBD	0.85	Eukaryota	Ascomycota	Saccharomycetes	Saccharomycetales	Saccharomycetaceae	Saccharomyces	v7
1000IBD00613	IBD	1	Eukaryota					Blastocystis	v7
1000IBD00682	IBD	1	Eukaryota					Blastocystis	v7
1000IBD00581	IBD	0.94	Eukaryota	Ascomycota	Saccharomycetes	Saccharomycetales	Saccharomycetaceae		v7
1000IBD00675	IBD	1	Eukaryota	Ascomycota	Saccharomycetes	Saccharomycetales			v7
1000IBD00679	IBD	0.94	Eukaryota	Ascomycota	Saccharomycetes	Saccharomycetales	Saccharomycetaceae		v7
SRR5127411	control	1	Eukaryota					Blastocystis	v4
SRR5127412	control	1	Eukaryota					Blastocystis	v4
SRR5127434	control	1	Eukaryota					Blastocystis	v4
SRR5127439	control	1	Eukaryota					Blastocystis	v4
SRR5127459	control	1	Eukaryota					Blastocystis	v4
SRR5127463	control	1	Eukaryota					Blastocystis	v4
SRR5127478	control	1	Eukaryota					Blastocystis	v4
SRR5127494	control	1	Eukaryota					Blastocystis	v4
SRR5127496	control	1	Eukaryota					Blastocystis	v4
SRR5127504	control	1	Eukaryota					Blastocystis	v4
SRR5127515	control	1	Eukaryota					Blastocystis	v4

SRR5127522	control	1	Eukaryota					Blastocystis	v4
SRR5127524	control	1	Eukaryota					Blastocystis	v4
SRR5127534	control	1	Eukaryota					Blastocystis	v4
SRR5127546	control	1	Eukaryota					Blastocystis	v4
SRR5127563	control	1	Eukaryota					Blastocystis	v4
SRR5127567	control	1	Eukaryota					Blastocystis	v4
SRR5127575	control	1	Eukaryota					Blastocystis	v4
SRR5127580	control	1	Eukaryota					Blastocystis	v4
SRR5127587	control	1	Eukaryota					Blastocystis	v4
SRR5127608	control	1	Eukaryota					Blastocystis	v4
SRR5127613	control	1	Eukaryota					Blastocystis	v4
SRR5127619	control	1	Eukaryota					Blastocystis	v4
SRR5127630	control	1	Eukaryota	Parabasalia	Tritrichomonadea			Dientamoeba	v4
SRR5127633	control	1	Eukaryota					Blastocystis	v4
SRR5127636	control	1	Eukaryota					Blastocystis	v4
SRR5127667	control	1	Eukaryota					Blastocystis	v4
SRR5127668	control	1	Eukaryota	Ascomycota	Saccharomycetes	Saccharomycetales	Saccharomycetaceae	Saccharomyces	v4
SRR5127688	control	1	Eukaryota					Blastocystis	v4
SRR5127688	control	1	Eukaryota	Parabasalia	Tritrichomonadea			Dientamoeba	v4
SRR5127717	control	1	Eukaryota					Blastocystis	v4
SRR5127731	control	1	Eukaryota					Blastocystis	v4
SRR5127737	control	1	Eukaryota					Blastocystis	v4
SRR5127752	control	1	Eukaryota	Parabasalia	Tritrichomonadea			Dientamoeba	v4
SRR5127754	control	1	Eukaryota					Blastocystis	v4
SRR5127754	control	1	Eukaryota					Blastocystis	v4
SRR5127772	control	1	Eukaryota					Blastocystis	v4

SRR5127782	control	1	Eukaryota					Blastocystis	v4
SRR5127783	control	1	Eukaryota					Blastocystis	v4
SRR5127793	control	1	Eukaryota					Blastocystis	v4
SRR5127796	control	1	Eukaryota					Blastocystis	v4
SRR5127823	control	1	Eukaryota					Blastocystis	v4
SRR5127841	control	1	Eukaryota					Blastocystis	v4
SRR5127847	control	1	Eukaryota					Blastocystis	v4
SRR5127850	control	1	Eukaryota					Blastocystis	v4
SRR5127857	control	1	Eukaryota					Blastocystis	v4
SRR5127397	control	1	Eukaryota					Blastocystis	v5
SRR5127411	control	1	Eukaryota					Blastocystis	v5
SRR5127412	control	1	Eukaryota					Blastocystis	v5
SRR5127417	control	0.9	Eukaryota	Ascomycota	Eurotiomycetes	Eurotiales	Trichocomaceae	Penicillium	v5
SRR5127455	control	1	Eukaryota					Blastocystis	v5
SRR5127455	control	1	Eukaryota	Parabasalia	Tritrichomonadea			Dientamoeba	v5
SRR5127463	control	1	Eukaryota					Blastocystis	v5
SRR5127476	control	1	Eukaryota					Blastocystis	v5
SRR5127478	control	1	Eukaryota					Blastocystis	v5
SRR5127494	control	1	Eukaryota					Blastocystis	v5
SRR5127496	control	1	Eukaryota					Blastocystis	v5
SRR5127504	control	1	Eukaryota					Blastocystis	v5
SRR5127514	control	1	Eukaryota					Blastocystis	v5
SRR5127514	control	1	Eukaryota					Blastocystis	v5
SRR5127522	control	1	Eukaryota					Blastocystis	v5
SRR5127524	control	1	Eukaryota	Parabasalia	Tritrichomonadea			Dientamoeba	v5
SRR5127546	control	1	Eukaryota					Blastocystis	v5
SRR5127567	control	1	Eukaryota					Blastocystis	v5

SRR5127580	control	1	Eukaryota					Blastocystis	v5
SRR5127587	control	1	Eukaryota					Blastocystis	v5
SRR5127608	control	1	Eukaryota					Blastocystis	v5
SRR5127613	control	1	Eukaryota					Blastocystis	v5
SRR5127619	control	1	Eukaryota					Blastocystis	v5
SRR5127620	control	1	Eukaryota					Blastocystis	v5
SRR5127621	control	1	Eukaryota					Blastocystis	v5
SRR5127630	control	1	Eukaryota					Blastocystis	v5
SRR5127630	control	1	Eukaryota	Parabasalia	Tritrichomonadea			Dientamoeba	v5
SRR5127633	control	1	Eukaryota					Blastocystis	v5
SRR5127636	control	1	Eukaryota					Blastocystis	v5
SRR5127636	control	1	Eukaryota	Parabasalia	Tritrichomonadea			Dientamoeba	v5
SRR5127642	control	1	Eukaryota	Parabasalia	Tritrichomonadea			Dientamoeba	v5
SRR5127667	control	1	Eukaryota					Blastocystis	v5
SRR5127679	control	1	Eukaryota					Blastocystis	v5
SRR5127688	control	1	Eukaryota	Parabasalia	Tritrichomonadea			Dientamoeba	v5
SRR5127688	control	1	Eukaryota					Blastocystis	v5
SRR5127715	control	1	Eukaryota					Blastocystis	v5
SRR5127717	control	1	Eukaryota					Blastocystis	v5
SRR5127719	control	1	Eukaryota	Parabasalia	Tritrichomonadea			Dientamoeba	v5
SRR5127737	control	1	Eukaryota					Blastocystis	v5
SRR5127752	control	1	Eukaryota	Parabasalia	Tritrichomonadea			Dientamoeba	v5
SRR5127754	control	1	Eukaryota					Blastocystis	v5
SRR5127756	control	1	Eukaryota	Parabasalia	Tritrichomonadea			Dientamoeba	v5

SRR5127756	control	1	Eukaryota					Blastocystis	v5
SRR5127770	control	1	Eukaryota					Blastocystis	v5
SRR5127773	control	1	Eukaryota					Blastocystis	v5
SRR5127774	control	1	Eukaryota					Blastocystis	v5
SRR5127782	control	1	Eukaryota					Blastocystis	v5
SRR5127783	control	1	Eukaryota					Blastocystis	v5
SRR5127798	control	1	Eukaryota					Blastocystis	v5
SRR5127801	control	1	Eukaryota					Blastocystis	v5
SRR5127815	control	1	Eukaryota	Parabasalia	Tritrichomonadea			Dientamoeba	v5
SRR5127834	control	1	Eukaryota	Parabasalia	Tritrichomonadea			Dientamoeba	v5
SRR5127841	control	1	Eukaryota	Parabasalia	Tritrichomonadea			Dientamoeba	v5
SRR5127842	control	1	Eukaryota					Blastocystis	v5
SRR5127844	control	1	Eukaryota					Blastocystis	v5
SRR5127847	control	1	Eukaryota					Blastocystis	v5
SRR5127850	control	1	Eukaryota					Blastocystis	v5
SRR5127857	control	1	Eukaryota					Blastocystis	v5
SRR5127858	control	1	Eukaryota					Blastocystis	v5
SRR5127439	control	1	Eukaryota					Blastocystis	v6
SRR5127459	control	1	Eukaryota						v6
SRR5127463	control	1	Eukaryota	Parabasalia	Tritrichomonadea			Dientamoeba	v6
SRR5127470	control	1	Eukaryota	Parabasalia	Tritrichomonadea			Dientamoeba	v6
SRR5127496	control	1	Eukaryota					Blastocystis	v6
SRR5127504	control	1	Eukaryota						v6
SRR5127524	control	1	Eukaryota	Parabasalia	Tritrichomonadea			Dientamoeba	v6
SRR5127546	control	1	Eukaryota					Blastocystis	v6

SRR5127587	control	1	Eukaryota						v6
SRR5127608	control	1	Eukaryota					Blastocystis	v6
SRR5127613	control	1	Eukaryota					Blastocystis	v6
SRR5127630	control	1	Eukaryota	Parabasalia	Tritrichomonadea			Dientamoeba	v6
SRR5127636	control	1	Eukaryota						v6
SRR5127636	control	1	Eukaryota	Parabasalia	Tritrichomonadea			Dientamoeba	v6
SRR5127667	control	1	Eukaryota					Blastocystis	v6
SRR5127688	control	1	Eukaryota	Parabasalia	Tritrichomonadea			Dientamoeba	v6
SRR5127707	control	1	Eukaryota						v6
SRR5127709	control	1	Eukaryota					Blastocystis	v6
SRR5127717	control	1	Eukaryota					Blastocystis	v6
SRR5127737	control	1	Eukaryota					Blastocystis	v6
SRR5127752	control	1	Eukaryota	Parabasalia	Tritrichomonadea			Dientamoeba	v6
SRR5127773	control	1	Eukaryota						v6
SRR5127773	control	1	Eukaryota	Parabasalia	Tritrichomonadea			Dientamoeba	v6
SRR5127782	control	1	Eukaryota						v6
SRR5127783	control	1	Eukaryota					Blastocystis	v6
SRR5127791	control	1	Eukaryota					Blastocystis	v6
SRR5127796	control	1	Eukaryota					Blastocystis	v6
SRR5127823	control	1	Eukaryota					Blastocystis	v6
SRR5127823	control	1	Eukaryota	Parabasalia	Tritrichomonadea			Dientamoeba	v6
SRR5127841	control	1	Eukaryota					Blastocystis	v6
SRR5127847	control	1	Eukaryota					Blastocystis	v6
SRR5127850	control	1	Eukaryota					Blastocystis	v6

SRR5127850	control	1	Eukaryota	Parabasalia	Tritrichomonadea			Dientamoeba	v6
SRR5127857	control	1	Eukaryota					Blastocystis	v6
SRR5127411	control	1	Eukaryota					Blastocystis	v7
SRR5127412	control	1	Eukaryota					Blastocystis	v7
SRR5127417	control	1	Eukaryota	Ascomycota	Eurotiomycetes	Eurotiales			v7
SRR5127455	control	1	Eukaryota					Blastocystis	v7
SRR5127457	control	1	Eukaryota					Blastocystis	v7
SRR5127459	control	1	Eukaryota					Blastocystis	v7
SRR5127463	control	1	Eukaryota					Blastocystis	v7
SRR5127476	control	1	Eukaryota					Blastocystis	v7
SRR5127478	control	1	Eukaryota					Blastocystis	v7
SRR5127494	control	1	Eukaryota					Blastocystis	v7
SRR5127504	control	1	Eukaryota					Blastocystis	v7
SRR5127514	control	1	Eukaryota					Blastocystis	v7
SRR5127514	control	1	Eukaryota					Blastocystis	v7
SRR5127522	control	1	Eukaryota					Blastocystis	v7
SRR5127524	control	1	Eukaryota					Blastocystis	v7
SRR5127537	control	1	Eukaryota					Blastocystis	v7
SRR5127546	control	1	Eukaryota					Blastocystis	v7
SRR5127580	control	1	Eukaryota					Blastocystis	v7
SRR5127587	control	1	Eukaryota					Blastocystis	v7
SRR5127608	control	1	Eukaryota					Blastocystis	v7
SRR5127608	control	1	Eukaryota	Parabasalia	Tritrichomonadea			Dientamoeba	v7
SRR5127613	control	1	Eukaryota					Blastocystis	v7
SRR5127621	control	1	Eukaryota					Blastocystis	v7
SRR5127630	control	1	Eukaryota					Blastocystis	v7

SRR5127630	control	1	Eukaryota	Parabasalia	Tritrichomonadea			Dientamoeba	v7
SRR5127633	control	1	Eukaryota					Blastocystis	v7
SRR5127636	control	1	Eukaryota					Blastocystis	v7
SRR5127636	control	1	Eukaryota	Parabasalia	Tritrichomonadea			Dientamoeba	v7
SRR5127651	control	0.85	Eukaryota	Ascomycota	Saccharomycetes	Saccharomycetales	Saccharomycetaceae	Saccharomyces	v7
SRR5127667	control	1	Eukaryota					Blastocystis	v7
SRR5127679	control	1	Eukaryota					Blastocystis	v7
SRR5127688	control	1	Eukaryota					Blastocystis	v7
SRR5127688	control	1	Eukaryota	Parabasalia	Tritrichomonadea			Dientamoeba	v7
SRR5127707	control	1	Eukaryota					Blastocystis	v7
SRR5127715	control	1	Eukaryota					Blastocystis	v7
SRR5127737	control	1	Eukaryota					Blastocystis	v7
SRR5127752	control	1	Eukaryota	Parabasalia	Tritrichomonadea			Dientamoeba	v7
SRR5127756	control	1	Eukaryota	Parabasalia	Tritrichomonadea			Dientamoeba	v7
SRR5127756	control	1	Eukaryota					Blastocystis	v7
SRR5127770	control	1	Eukaryota					Blastocystis	v7
SRR5127772	control	1	Eukaryota					Blastocystis	v7
SRR5127773	control	1	Eukaryota					Blastocystis	v7
SRR5127774	control	1	Eukaryota					Blastocystis	v7
SRR5127782	control	1	Eukaryota	Parabasalia	Tritrichomonadea			Dientamoeba	v7
SRR5127783	control	1	Eukaryota					Blastocystis	v7
SRR5127783	control	1	Eukaryota	Fornicata	Diplomonadida	Giardiinae		Giardia	v7
SRR5127791	control	1	Eukaryota					Blastocystis	v7

SRR5127823	control	1	Eukaryota					Blastocystis	v7
SRR5127834	control	1	Eukaryota	Parabasalia	Tritrichomonadea			Dientamoeba	v7
SRR5127841	control	1	Eukaryota					Blastocystis	v7
SRR5127844	control	1	Eukaryota					Blastocystis	v7
SRR5127847	control	1	Eukaryota					Blastocystis	v7
SRR5127850	control	1	Eukaryota					Blastocystis	v7
SRR5127854	control	1	Eukaryota					Blastocystis	v7
SRR5127857	control	1	Eukaryota					Blastocystis	v7
SRR5127858	control	1	Eukaryota					Blastocystis	v7

Table S2. Results of EukDetect for the 1000IBD and 500FG studies before rarefying.

Sample ID	Taxa	Taxa ID	Observed markers	Read counts	Percent observed markers	Total marker coverage	Percent identity
1000IBD00010	Saccharomyces cerevisiae S288C	559292	55	145	30.22%	7.88%	99.57%
1000IBD00014	Candida albicans SC5314	237561	11	26	6.55%	9.66%	100.00%
1000IBD00016	Saccharomyces cerevisiae S288C	559292	11	19	6.04%	8.64%	99.80%
1000IBD00035	Saccharomyces cerevisiae S288C	559292	4	8	2.20%	4.80%	100.00%
1000IBD00035	Clavispora lusitaniae ATCC 42720	306902	2	4	1.16%	2.68%	99.58%
1000IBD00041	Saccharomyces cerevisiae S288C	559292	6	11	3.30%	3.79%	99.19%
1000IBD00046	Saccharomyces cerevisiae S288C	559292	2	4	1.10%	8.79%	98.90%
1000IBD00050	Candida glabrata	5478	5	12	3.12%	5.83%	99.87%
1000IBD00053	Saccharomyces cerevisiae S288C	559292	5	11	2.75%	6.74%	99.64%
1000IBD00058	Saccharomyces cerevisiae S288C	559292	6	10	3.30%	4.04%	99.64%
1000IBD00067	Saccharomyces cerevisiae S288C	559292	4	12	2.20%	9.40%	99.63%
1000IBD00067	Candida albicans SC5314	237561	3	7	1.79%	7.94%	99.84%
1000IBD00084	Saccharomyces cerevisiae S288C	559292	2	4	1.10%	3.30%	100.00%
1000IBD00103	Saccharomyces cerevisiae S288C	559292	8	16	4.40%	4.67%	99.91%
1000IBD00107	Saccharomyces cerevisiae S288C	559292	120	520	65.93%	13.52%	99.68%
1000IBD00114	Saccharomyces cerevisiae S288C	559292	29	63	15.93%	7.71%	99.68%
1000IBD00122	Saccharomyces cerevisiae S288C	559292	3	6	1.65%	3.80%	99.71%
1000IBD00154	Saccharomyces cerevisiae S288C	559292	2	4	1.10%	8.36%	99.45%
1000IBD00155	Saccharomyces cerevisiae S288C	559292	30	65	16.48%	7.19%	99.56%
1000IBD00157	Malassezia restricta	76775	3	6	2.00%	1.71%	99.49%
1000IBD00173	Penicillium roqueforti FM164	1365484	6	11	3.02%	5.70%	100.00%
1000IBD00187	Saccharomyces cerevisiae S288C	559292	18	38	9.89%	4.92%	99.61%
1000IBD00188	Saccharomyces cerevisiae S288C	559292	20	44	10.99%	5.31%	99.68%

1000IBD00197	Saccharomyces cerevisiae S288C	559292	2	4	1.10%	2.59%	100.00%
1000IBD00198	Blastocystis sp. subtype 4	944170	8	31	80.00%	13.44%	99.77%
1000IBD00202	Blastocystis hominis	12968	3	10	23.08%	12.27%	99.07%
1000IBD00204	Saccharomyces cerevisiae S288C	559292	4	8	2.20%	3.42%	99.68%
1000IBD00206	Blastocystis sp. subtype 2	944160	7	24	33.33%	9.89%	99.13%
1000IBD00208	Blastocystis sp. subtype 4	944170	7	29	70.00%	17.13%	99.78%
1000IBD00225	Saccharomyces cerevisiae S288C	559292	37	87	20.33%	6.01%	99.76%
1000IBD00228	Saccharomyces cerevisiae S288C	559292	5	9	2.75%	4.16%	99.73%
1000IBD00239	Blastocystis sp. ATCC 50177/Nand II	478820	7	21	35.00%	9.51%	99.25%
1000IBD00255	Candida albicans SC5314	237561	2	4	1.19%	5.88%	100.00%
1000IBD00266	Saccharomyces cerevisiae S288C	559292	3	6	1.65%	9.59%	100.00%
1000IBD00277	Saccharomyces cerevisiae S288C	559292	3	6	1.65%	4.52%	99.80%
1000IBD00281	Saccharomyces cerevisiae S288C	559292	4	7	2.20%	6.00%	100.00%
1000IBD00301	Candida glabrata	5478	9	19	5.62%	5.10%	99.73%
1000IBD00304	Saccharomyces cerevisiae S288C	559292	5	9	2.75%	4.13%	99.64%
1000IBD00310	Saccharomyces cerevisiae S288C	559292	2	4	1.10%	5.93%	100.00%
1000IBD00329	Saccharomyces cerevisiae S288C	559292	2	4	1.10%	3.19%	99.61%
1000IBD00339	Candida glabrata	5478	5	12	3.12%	8.20%	99.54%
1000IBD00342	Blastocystis sp. subtype 3	944168	14	53	63.64%	13.04%	99.44%
1000IBD00345	Saccharomyces cerevisiae S288C	559292	13	24	7.14%	7.19%	99.40%
1000IBD00349	Blastocystis sp. subtype 4	944170	10	66	100.00%	26.83%	99.77%
1000IBD00367	Saccharomyces cerevisiae S288C	559292	4	7	2.20%	7.89%	99.59%
1000IBD00370	Saccharomyces cerevisiae S288C	559292	3	7	1.65%	5.92%	99.63%
1000IBD00389	Candida glabrata	5478	5	11	3.12%	12.47%	99.77%
1000IBD00401	Saccharomyces cerevisiae S288C	559292	52	137	28.57%	7.93%	99.74%
1000IBD00401	Candida glabrata	5478	26	61	16.25%	8.10%	99.75%
1000IBD00416	Saccharomyces cerevisiae S288C	559292	3	6	1.65%	11.49%	98.06%
1000IBD00419	Candida glabrata	5478	17	45	10.62%	5.91%	99.65%

1000IBD00427	Blastocystis sp. subtype 9	1544353	6	29	54.55%	16.05%	99.60%
1000IBD00431	Blastocystis sp. subtype 2	944160	15	82	71.43%	19.69%	98.81%
1000IBD00433	Blastocystis sp. subtype 3	944168	5	7	22.73%	6.75%	99.39%
1000IBD00442	Penicillium roqueforti FM164	1365484	7	15	3.52%	5.60%	100.00%
1000IBD00469	Saccharomyces cerevisiae S288C	559292	3	6	1.65%	4.07%	99.75%
1000IBD00470	Candida glabrata	5478	25	59	15.62%	9.12%	99.62%
1000IBD00470	Candida albicans SC5314	237561	2	4	1.19%	10.34%	99.50%
1000IBD00485	Saccharomyces cerevisiae S288C	559292	2	4	1.10%	4.52%	100.00%
1000IBD00495	Saccharomyces cerevisiae S288C	559292	2	4	1.10%	7.23%	100.00%
1000IBD00502	Saccharomyces cerevisiae S288C	559292	4	7	2.20%	7.23%	100.00%
1000IBD00505	Saccharomyces cerevisiae S288C	559292	4	8	2.20%	2.56%	99.35%
1000IBD00510	Blastocystis sp. subtype 2	944160	21	300	100.00%	43.00%	98.72%
1000IBD00511	Saccharomyces cerevisiae S288C	559292	5	10	2.75%	5.68%	99.53%
1000IBD00512	Saccharomyces cerevisiae S288C	559292	41	95	22.53%	7.39%	99.69%
1000IBD00514	Blastocystis sp. subtype 3	944168	8	34	36.36%	13.58%	99.69%
1000IBD00535	Saccharomyces cerevisiae S288C	559292	8	20	4.40%	6.10%	99.36%
1000IBD00535	Candida albicans SC5314	237561	2	4	1.19%	4.25%	100.00%
1000IBD00541	Saccharomyces cerevisiae S288C	559292	6	12	3.30%	4.49%	100.00%
1000IBD00550	Candida glabrata	5478	46	109	28.75%	9.09%	99.58%
1000IBD00550	Saccharomyces cerevisiae S288C	559292	46	106	25.27%	7.72%	99.73%
1000IBD00556	Saccharomyces cerevisiae S288C	559292	128	582	70.33%	15.50%	99.66%
1000IBD00563	Penicillium roqueforti FM164	1365484	3	6	1.51%	5.29%	100.00%
1000IBD00565	Saccharomyces cerevisiae S288C	559292	2	4	1.10%	5.89%	99.66%
1000IBD00569	Saccharomyces cerevisiae S288C	559292	8	18	4.40%	4.07%	99.68%
1000IBD00586	Saccharomyces cerevisiae S288C	559292	16	31	8.79%	5.78%	99.61%
1000IBD00593	Saccharomyces cerevisiae S288C	559292	5	10	2.75%	3.10%	99.37%

1000IBD00593	Meyerozyma	766728	3	6	1.94%	8.20%	100.00%
1000IBD00593	Wickerhamomyces anomalus NRRL Y-366-8	683960	2	4	1.14%	8.86%	97.66%
1000IBD00598	Debaryomyces hansenii CBS767	284592	6	13	3.28%	4.25%	99.50%
1000IBD00598	Saccharomyces cerevisiae S288C	559292	2	4	1.10%	4.13%	99.48%
1000IBD00610	Saccharomyces cerevisiae S288C	559292	5	10	2.75%	5.52%	99.56%
1000IBD00613	Blastocystis sp. subtype 2	944160	10	28	47.62%	7.75%	99.19%
1000IBD00624	Saccharomyces cerevisiae S288C	559292	9	15	4.95%	3.16%	99.60%
1000IBD00627	Penicillium roqueforti FM164	1365484	4	10	2.01%	6.59%	100.00%
1000IBD00644	Blastocystis sp. subtype 3	944168	7	20	31.82%	7.35%	99.67%
1000IBD00645	Saccharomyces cerevisiae S288C	559292	4	7	2.20%	4.88%	99.85%
1000IBD00646	Blastocystis sp. subtype 3	944168	6	12	27.27%	5.88%	99.67%
1000IBD00649	Saccharomyces cerevisiae S288C	559292	5	10	2.75%	3.88%	99.87%
1000IBD00670	Saccharomyces cerevisiae S288C	559292	7	14	3.85%	4.72%	100.00%
1000IBD00676	Debaryomyces hansenii CBS767	284592	10	25	5.46%	13.26%	99.95%
1000IBD00682	Blastocystis sp. subtype 3	944168	10	36	45.45%	9.44%	99.84%
1000IBD00682	Saccharomyces cerevisiae S288C	559292	25	46	13.74%	7.44%	99.80%
1000IBD00698	Saccharomyces cerevisiae S288C	559292	25	49	13.74%	5.02%	99.71%
1000IBD00385	Saccharomyces cerevisiae S288C	559292	4	7	2.20%	6.64%	99.80%
1000IBD00394	Debaryomyces hansenii CBS767	284592	7	13	3.83%	5.18%	99.77%
1000IBD00476	Blastocystis sp. subtype 4	944170	9	51	90.00%	19.76%	99.82%
1000IBD00480	Blastocystis sp. subtype 2	944160	10	24	47.62%	7.45%	98.98%
1000IBD00487	Saccharomyces cerevisiae S288C	559292	13	31	7.14%	6.07%	99.33%
1000IBD00581	Saccharomyces cerevisiae S288C	559292	74	198	40.66%	7.69%	99.52%
1000IBD00581	Candida albicans SC5314	237561	38	81	22.62%	7.17%	99.63%
1000IBD00614	Saccharomyces cerevisiae S288C	559292	2	4	1.10%	6.59%	100.00%

1000IBD00675	Debaryomyces hansenii CBS767	284592	33	67	18.03%	5.19%	99.46%
1000IBD00679	Saccharomyces cerevisiae S288C	559292	39	79	21.43%	6.93%	99.70%
1000IBD00680	Blastocystis sp. subtype 4	944170	2	4	20.00%	6.28%	100.00%
1000IBD00705	Saccharomyces cerevisiae S288C	559292	2	5	1.10%	2.74%	99.74%
1000IBD00708	Saccharomyces cerevisiae S288C	559292	6	12	3.30%	8.43%	99.69%
1000IBD00715	Saccharomyces cerevisiae S288C	559292	23	51	12.64%	5.18%	99.51%
1000IBD00720	Cyberlindnera jadinii NRRL Y-1542	983966	10	20	5.78%	4.60%	99.62%
1000IBD01328	Saccharomyces cerevisiae S288C	559292	14	26	7.69%	7.56%	99.90%
1000IBD01329	Blastocystis sp. subtype 3	944168	9	28	40.91%	8.94%	99.53%
SRR5127393	Blastocystis sp. subtype 2	944160	4	7	19.05%	5.57%	99.11%
SRR5127394	Saccharomyces cerevisiae S288C	559292	2	4	1.10%	8.86%	98.78%
SRR5127397	Blastocystis sp. subtype 2	944160	16	83	76.19%	14.63%	98.90%
SRR5127400	Blastocystis sp. subtype 3	944168	15	61	68.18%	10.92%	99.56%
SRR5127406	Blastocystis sp. subtype 3	944168	15	62	68.18%	11.34%	99.45%
SRR5127407	Blastocystis sp. subtype 3	944168	8	18	36.36%	6.00%	99.17%
SRR5127408	Blastocystis sp. subtype 3	944168	4	15	18.18%	6.50%	99.29%
SRR5127411	Blastocystis sp. ATCC 50177/Nand II	478820	18	136	90.00%	25.39%	98.97%
SRR5127411	Saccharomyces cerevisiae S288C	559292	2	4	1.10%	8.47%	100.00%
SRR5127412	Blastocystis sp. ATCC 50177/Nand II	478820	14	64	70.00%	15.93%	99.15%
SRR5127417	Penicillium roqueforti FM164	1365484	59	138	29.65%	7.27%	99.97%
SRR5127422	Saccharomyces cerevisiae S288C	559292	8	15	4.40%	7.27%	99.76%
SRR5127425	Blastocystis sp. subtype 3	944168	7	17	31.82%	5.22%	99.46%
SRR5127425	Saccharomyces cerevisiae S288C	559292	7	12	3.85%	5.04%	99.59%
SRR5127425	Penicillium roqueforti FM164	1365484	2	4	1.01%	5.37%	100.00%
SRR5127434	Blastocystis sp. subtype 2	944160	10	34	47.62%	10.47%	99.19%

SRR5127434	Giardia lamblia ATCC 50803	184922	2	4	10.00%	8.77%	98.13%
SRR5127437	Saccharomyces cerevisiae S288C	559292	4	7	2.20%	4.05%	99.78%
SRR5127439	Blastocystis sp. subtype 4	944170	5	17	50.00%	12.16%	100.00%
SRR5127439	Saccharomyces cerevisiae S288C	559292	5	15	2.75%	6.22%	99.81%
SRR5127443	Blastocystis sp. subtype 2	944160	5	13	23.81%	6.79%	98.65%
SRR5127452	Saccharomyces cerevisiae S288C	559292	12	20	6.59%	5.81%	99.47%
SRR5127453	Saccharomyces cerevisiae S288C	559292	4	8	2.20%	3.93%	99.38%
SRR5127454	Saccharomyces cerevisiae S288C	559292	3	4	1.65%	1.95%	100.00%
SRR5127455	Blastocystis sp. subtype 2	944160	15	53	71.43%	12.61%	98.74%
SRR5127456	Blastocystis sp. subtype 3	944168	13	49	59.09%	9.33%	99.60%
SRR5127457	Blastocystis sp. ATCC 50177/Nand II	478820	13	51	65.00%	12.84%	99.44%
SRR5127458	Saccharomyces cerevisiae S288C	559292	7	12	3.85%	9.28%	99.69%
SRR5127459	Blastocystis sp. subtype 2	944160	16	62	76.19%	13.59%	98.96%
SRR5127461	Debaryomyces hansenii CBS767	284592	4	8	2.19%	3.79%	100.00%
SRR5127461	Candida sake	39397	3	5	1.67%	4.14%	99.26%
SRR5127463	Blastocystis sp. subtype 2	944160	15	101	71.43%	23.45%	98.87%
SRR5127466	Penicillium roqueforti FM164	1365484	5	9	2.51%	3.57%	100.00%
SRR5127469	Malassezia restricta	76775	2	4	1.33%	1.53%	98.89%
SRR5127470	Blastocystis sp. subtype 3	944168	11	63	50.00%	10.95%	99.62%
SRR5127470	Saccharomyces cerevisiae S288C	559292	27	56	14.84%	5.83%	99.76%
SRR5127471	Blastocystis sp. subtype 4	944170	6	17	60.00%	7.72%	99.55%
SRR5127476	Blastocystis sp. subtype 2	944160	17	110	80.95%	22.57%	98.90%
SRR5127478	Blastocystis sp. subtype 3	944168	19	193	86.36%	28.33%	99.46%
SRR5127480	Blastocystis sp. subtype 3	944168	4	9	18.18%	5.04%	99.86%
SRR5127489	Blastocystis sp. subtype 2	944160	2	6	9.52%	3.93%	97.22%
SRR5127490	Saccharomyces cerevisiae S288C	559292	5	11	2.75%	5.39%	99.67%

SRR5127492	Saccharomyces cerevisiae S288C	559292	5	9	2.75%	4.91%	99.81%
SRR5127494	Blastocystis sp. subtype 2	944160	19	218	90.48%	37.42%	98.93%
SRR5127495	Blastocystis sp. subtype 3	944168	4	8	18.18%	4.18%	99.61%
SRR5127496	Blastocystis sp. subtype 4	944170	10	59	100.00%	19.27%	99.65%
SRR5127499	Saccharomyces cerevisiae S288C	559292	8	15	4.40%	2.54%	99.50%
SRR5127504	Blastocystis sp. subtype 2	944160	20	178	95.24%	27.60%	99.01%
SRR5127505	Blastocystis sp. subtype 2	944160	7	21	33.33%	9.59%	99.17%
SRR5127514	Blastocystis sp. subtype 3	944168	14	38	63.64%	8.13%	99.01%
SRR5127514	Blastocystis sp. ATCC 50177/Nand II	478820	12	41	60.00%	10.46%	98.98%
SRR5127515	Blastocystis sp. subtype 4	944170	8	44	80.00%	20.42%	99.97%
SRR5127516	Blastocystis sp. subtype 3	944168	3	6	13.64%	3.35%	99.21%
SRR5127519	Blastocystis sp. subtype 3	944168	9	42	40.91%	10.47%	99.78%
SRR5127520	Saccharomyces cerevisiae S288C	559292	6	10	3.30%	9.51%	99.34%
SRR5127522	Blastocystis sp. subtype 2	944160	20	142	95.24%	24.82%	98.84%
SRR5127523	Blastocystis sp. subtype 3	944168	15	72	68.18%	13.31%	99.46%
SRR5127524	Blastocystis sp. ATCC 50177/Nand II	478820	20	268	100.00%	38.03%	99.19%
SRR5127525	Blastocystis sp. subtype 3	944168	16	101	72.73%	15.37%	99.39%
SRR5127529	Blastocystis sp. subtype 3	944168	5	24	22.73%	11.23%	99.54%
SRR5127534	Blastocystis sp. subtype 2	944160	15	52	71.43%	12.76%	98.89%
SRR5127537	Blastocystis sp. subtype 3	944168	12	55	54.55%	10.53%	99.52%
SRR5127539	Blastocystis sp. subtype 3	944168	3	7	13.64%	3.55%	100.00%
SRR5127541	Blastocystis sp. subtype 2	944160	11	28	52.38%	8.67%	98.79%
SRR5127543	Blastocystis sp. subtype 2	944160	2	6	9.52%	6.92%	100.00%
SRR5127544	Giardia lamblia ATCC 50803	184922	4	12	20.00%	8.49%	99.22%
SRR5127546	Blastocystis sp. subtype 4	944170	10	64	100.00%	22.32%	99.87%

SRR5127548	Penicillium roqueforti FM164	1365484	10	17	5.03%	6.12%	100.00%
SRR5127548	Saccharomyces cerevisiae S288C	559292	2	4	1.10%	6.17%	100.00%
SRR5127551	Saccharomyces cerevisiae S288C	559292	2	5	1.10%	4.24%	100.00%
SRR5127552	Saccharomyces cerevisiae S288C	559292	4	6	2.20%	3.51%	99.56%
SRR5127556	Blastocystis sp. subtype 3	944168	3	9	13.64%	6.13%	99.83%
SRR5127557	Blastocystis sp. subtype 2	944160	9	27	42.86%	7.61%	98.57%
SRR5127563	Blastocystis sp. subtype 2	944160	11	47	52.38%	10.31%	98.73%
SRR5127565	Blastocystis sp. subtype 2	944160	4	6	19.05%	8.25%	98.48%
SRR5127566	Blastocystis sp. subtype 2	944160	8	13	38.10%	5.02%	98.68%
SRR5127567	Blastocystis sp. ATCC 50177/Nand II	478820	20	170	100.00%	28.40%	99.12%
SRR5127575	Blastocystis sp. subtype 4	944170	9	38	90.00%	12.83%	99.82%
SRR5127576	Blastocystis sp. ATCC 50177/Nand II	478820	3	6	15.00%	2.87%	99.12%
SRR5127579	Blastocystis sp. subtype 3	944168	4	11	18.18%	4.60%	99.21%
SRR5127580	Blastocystis sp. subtype 2	944160	18	129	85.71%	24.67%	99.05%
SRR5127581	Blastocystis sp. ATCC 50177/Nand II	478820	6	19	30.00%	6.21%	99.37%
SRR5127585	Blastocystis sp. ATCC 50177/Nand II	478820	4	7	20.00%	5.90%	99.04%
SRR5127587	Blastocystis sp. subtype 2	944160	20	164	95.24%	28.61%	98.88%
SRR5127588	Blastocystis sp. subtype 3	944168	8	18	36.36%	7.16%	98.90%
SRR5127589	Hanseniaspora uvarum	29833	3	7	2.61%	4.25%	100.00%
SRR5127590	Blastocystis sp. subtype 3	944168	2	4	9.09%	7.35%	99.38%
SRR5127597	Blastocystis sp. subtype 3	944168	6	15	27.27%	5.30%	99.49%
SRR5127599	Saccharomyces cerevisiae S288C	559292	5	9	2.75%	4.90%	99.64%
SRR5127600	Saccharomyces cerevisiae S288C	559292	16	31	8.79%	4.85%	99.63%
SRR5127602	Blastocystis sp. subtype 2	944160	11	25	52.38%	7.27%	99.04%
SRR5127603	Blastocystis sp. subtype 2	944160	7	16	33.33%	5.64%	99.10%

SRR5127608	Blastocystis sp. subtype 3	944168	22	527	100.00%	53.74%	99.42%
SRR5127613	Blastocystis sp. subtype 4	944170	10	98	100.00%	30.15%	99.85%
SRR5127613	Saccharomyces cerevisiae S288C	559292	3	7	1.65%	4.47%	99.73%
SRR5127618	Saccharomyces cerevisiae S288C	559292	4	9	2.20%	8.33%	99.85%
SRR5127619	Blastocystis sp. ATCC 50177/Nand II	478820	10	28	50.00%	11.45%	99.43%
SRR5127620	Blastocystis sp. ATCC 50177/Nand II	478820	11	30	55.00%	8.53%	99.13%
SRR5127621	Blastocystis sp. subtype 4	944170	7	41	70.00%	16.44%	99.96%
SRR5127622	Blastocystis sp. ATCC 50177/Nand II	478820	5	11	25.00%	5.48%	99.68%
SRR5127627	Blastocystis sp. subtype 3	944168	10	32	45.45%	8.72%	99.68%
SRR5127627	Penicillium roqueforti FM164	1365484	4	7	2.01%	7.70%	99.84%
SRR5127628	Blastocystis sp. subtype 4	944170	2	4	20.00%	4.64%	99.52%
SRR5127629	Saccharomyces cerevisiae S288C	559292	6	9	3.30%	8.25%	99.72%
SRR5127630	Blastocystis sp. subtype 2	944160	21	282	100.00%	35.11%	99.02%
SRR5127633	Blastocystis sp. subtype 3	944168	19	153	86.36%	23.98%	99.54%
SRR5127634	Debaryomyces hansenii CBS767	284592	2	4	1.09%	2.74%	100.00%
SRR5127634	Penicillium roqueforti FM164	1365484	2	4	1.01%	7.12%	100.00%
SRR5127636	Blastocystis sp. ATCC 50177/Nand II	478820	20	503	100.00%	56.98%	99.00%
SRR5127637	Blastocystis sp. subtype 2	944160	6	16	28.57%	9.15%	98.95%
SRR5127642	Blastocystis sp. subtype 3	944168	14	70	63.64%	12.59%	99.51%
SRR5127643	Blastocystis sp. ATCC 50177/Nand II	478820	5	12	25.00%	5.78%	99.78%
SRR5127643	Saccharomyces cerevisiae S288C	559292	26	52	14.29%	5.43%	99.53%
SRR5127648	Blastocystis sp. ATCC 50177/Nand II	478820	6	12	30.00%	4.10%	99.00%
SRR5127649	Saccharomyces cerevisiae S288C	559292	2	4	1.10%	13.20%	100.00%
SRR5127651	Saccharomyces cerevisiae S288C	559292	51	121	28.02%	7.50%	99.70%
SRR5127655	Blastocystis sp. subtype 2	944160	16	56	76.19%	11.16%	98.89%

SRR5127657	Saccharomyces cerevisiae S288C	559292	3	5	1.65%	9.75%	100.00%
SRR5127667	Blastocystis sp. subtype 4	944170	8	125	80.00%	40.82%	99.73%
SRR5127667	Penicillium roqueforti FM164	1365484	4	7	2.01%	8.95%	100.00%
SRR5127672	Pichia kudriavzevii	4909	11	36	6.96%	6.85%	99.85%
SRR5127679	Blastocystis sp. subtype 2	944160	14	71	66.67%	14.98%	99.17%
SRR5127681	Blastocystis sp. subtype 3	944168	2	4	9.09%	5.85%	98.76%
SRR5127685	Blastocystis sp. subtype 2	944160	10	19	47.62%	5.25%	98.93%
SRR5127688	Blastocystis sp. subtype 3	944168	20	210	90.91%	29.74%	99.37%
SRR5127691	Saccharomyces cerevisiae S288C	559292	4	7	2.20%	8.00%	99.80%
SRR5127698	Saccharomyces cerevisiae S288C	559292	2	4	1.10%	5.75%	100.00%
SRR5127707	Blastocystis sp. subtype 2	944160	17	62	80.95%	13.92%	99.01%
SRR5127709	Blastocystis sp. subtype 4	944170	9	46	90.00%	19.06%	99.84%
SRR5127710	Blastocystis sp. ATCC 50177/Nand II	478820	12	36	60.00%	10.20%	99.35%
SRR5127712	Blastocystis sp. ATCC 50177/Nand II	478820	4	10	20.00%	7.10%	99.68%
SRR5127715	Blastocystis sp. subtype 2	944160	18	115	85.71%	22.74%	99.00%
SRR5127717	Blastocystis sp. subtype 3	944168	20	212	90.91%	32.46%	99.46%
SRR5127719	Blastocystis sp. subtype 4	944170	7	23	70.00%	9.65%	99.85%
SRR5127722	Blastocystis sp. subtype 2	944160	4	10	19.05%	6.72%	99.73%
SRR5127724	Blastocystis sp. subtype 2	944160	7	13	33.33%	7.02%	98.85%
SRR5127728	Saccharomyces cerevisiae S288C	559292	18	43	9.89%	6.15%	99.73%
SRR5127731	Blastocystis sp. ATCC 50177/Nand II	478820	6	13	30.00%	5.16%	99.38%
SRR5127731	Saccharomyces cerevisiae S288C	559292	3	5	1.65%	3.05%	99.61%
SRR5127736	Malassezia restricta	76775	2	4	1.33%	4.59%	99.45%
SRR5127737	Blastocystis sp. subtype 3	944168	22	290	100.00%	36.11%	99.32%
SRR5127738	Blastocystis sp. ATCC 50177/Nand II	478820	8	24	40.00%	7.01%	98.57%
SRR5127739	Saccharomyces cerevisiae S288C	559292	32	82	17.58%	6.13%	99.70%

SRR5127744	Debaryomyces hansenii CBS767	284592	9	20	4.92%	5.69%	99.79%
SRR5127751	Penicillium roqueforti FM164	1365484	5	10	2.51%	6.81%	100.00%
SRR5127752	Blastocystis sp. subtype 3	944168	2	4	9.09%	3.03%	99.46%
SRR5127754	Blastocystis sp. subtype 3	944168	21	163	95.45%	24.69%	99.41%
SRR5127754	Blastocystis sp. ATCC 50177/Nand II	478820	11	43	55.00%	11.69%	99.03%
SRR5127754	Saccharomyces cerevisiae S288C	559292	5	10	2.75%	4.28%	100.00%
SRR5127756	Blastocystis sp. subtype 3	944168	18	140	81.82%	22.98%	99.50%
SRR5127764	Blastocystis sp. subtype 2	944160	8	28	38.10%	9.16%	98.18%
SRR5127769	Blastocystis sp. subtype 3	944168	4	7	18.18%	4.31%	99.65%
SRR5127770	Blastocystis sp. subtype 2	944160	18	84	85.71%	16.68%	98.88%
SRR5127772	Blastocystis sp. subtype 2	944160	16	108	76.19%	21.80%	99.02%
SRR5127773	Blastocystis sp. subtype 2	944160	21	361	100.00%	47.78%	98.75%
SRR5127774	Blastocystis sp. ATCC 50177/Nand II	478820	17	57	85.00%	13.33%	99.27%
SRR5127776	Blastocystis sp. subtype 3	944168	3	6	13.64%	3.07%	99.68%
SRR5127778	Cyberlindnera jadinii NRRL Y-1542	983966	3	6	1.73%	4.02%	99.25%
SRR5127780	Blastocystis sp. subtype 2	944160	6	23	28.57%	8.35%	99.50%
SRR5127782	Blastocystis sp. ATCC 50177/Nand II	478820	15	140	75.00%	25.93%	99.32%
SRR5127783	Blastocystis sp. subtype 3	944168	22	369	100.00%	43.53%	99.37%
SRR5127783	Giardia lamblia ATCC 50803	184922	9	29	45.00%	11.72%	98.97%
SRR5127788	Saccharomyces cerevisiae S288C	559292	3	6	1.65%	3.51%	100.00%
SRR5127791	Blastocystis sp. subtype 3	944168	19	201	86.36%	27.46%	99.43%
SRR5127791	Saccharomyces cerevisiae S288C	559292	2	4	1.10%	9.20%	99.32%
SRR5127793	Blastocystis sp. subtype 4	944170	8	34	80.00%	11.72%	99.79%
SRR5127793	Saccharomyces cerevisiae S288C	559292	2	4	1.10%	3.21%	100.00%
SRR5127796	Blastocystis sp. subtype 3	944168	17	173	77.27%	24.70%	99.24%

SRR5127798	Blastocystis sp. subtype 2	944160	11	38	52.38%	11.25%	99.22%
SRR5127801	Blastocystis sp. subtype 2	944160	6	13	28.57%	6.92%	98.89%
SRR5127803	Blastocystis sp. subtype 3	944168	4	8	18.18%	7.06%	99.11%
SRR5127806	Blastocystis sp. ATCC 50177/Nand II	478820	5	10	25.00%	3.34%	99.42%
SRR5127810	Blastocystis sp. subtype 4	944170	5	11	50.00%	6.58%	100.00%
SRR5127810	Saccharomyces cerevisiae S288C	559292	11	22	6.04%	4.07%	99.87%
SRR5127814	Blastocystis sp. subtype 4	944170	2	5	20.00%	5.15%	100.00%
SRR5127815	Blastocystis sp. subtype 2	944160	9	24	42.86%	8.07%	98.49%
SRR5127815	Debaryomyces hansenii CBS767	284592	9	22	4.92%	5.37%	99.80%
SRR5127816	Blastocystis sp. subtype 3	944168	2	4	9.09%	2.70%	100.00%
SRR5127819	Blastocystis sp. subtype 2	944160	5	9	23.81%	7.08%	98.83%
SRR5127821	Cyberlindnera jadinii NRRL Y-1542	983966	12	21	6.94%	5.30%	99.25%
SRR5127821	Saccharomyces cerevisiae S288C	559292	11	22	6.04%	4.33%	99.54%
SRR5127823	Blastocystis sp. subtype 3	944168	21	233	95.45%	32.19%	99.55%
SRR5127823	Pichia membranifaciens NRRL Y-2026	763406	2	4	1.20%	21.17%	99.59%
SRR5127823	Saccharomyces cerevisiae S288C	559292	2	4	1.10%	1.71%	99.40%
SRR5127825	Saccharomyces cerevisiae S288C	559292	3	6	1.65%	1.57%	99.62%
SRR5127828	Blastocystis sp. ATCC 50177/Nand II	478820	12	44	60.00%	11.30%	99.47%
SRR5127829	Saccharomyces cerevisiae S288C	559292	5	10	2.75%	2.74%	99.65%
SRR5127831	Blastocystis sp. subtype 2	944160	13	56	61.90%	12.77%	99.03%
SRR5127832	Saccharomyces cerevisiae S288C	559292	4	7	2.20%	4.76%	99.81%
SRR5127834	Pichia fermentans	53655	3	6	2.04%	3.38%	99.54%
SRR5127841	Blastocystis sp. subtype 3	944168	20	166	90.91%	21.25%	99.43%
SRR5127841	Saccharomyces cerevisiae S288C	559292	5	9	2.75%	3.01%	99.79%
SRR5127842	Blastocystis sp. subtype 2	944160	16	61	76.19%	13.84%	99.06%

SRR5127844	Blastocystis sp. ATCC 50177/Nand II	478820	18	124	90.00%	22.92%	99.32%
SRR5127845	Blastocystis sp. subtype 3	944168	6	11	27.27%	4.90%	99.14%
SRR5127847	Blastocystis sp. subtype 3	944168	20	390	90.91%	47.24%	99.45%
SRR5127847	Saccharomyces cerevisiae S288C	559292	8	19	4.40%	6.42%	99.93%
SRR5127848	Blastocystis sp. subtype 3	944168	4	13	18.18%	6.66%	99.20%
SRR5127848	Blastocystis sp. ATCC 50177/Nand II	478820	3	10	15.00%	6.59%	99.28%
SRR5127850	Blastocystis sp. subtype 4	944170	10	134	100.00%	40.80%	99.71%
SRR5127854	Blastocystis sp. subtype 2	944160	11	30	52.38%	8.88%	99.22%
SRR5127854	Saccharomyces cerevisiae S288C	559292	3	5	1.65%	7.64%	100.00%
SRR5127855	Blastocystis sp. subtype 2	944160	11	44	52.38%	10.22%	99.10%
SRR5127857	Blastocystis sp. subtype 3	944168	22	269	100.00%	33.57%	99.46%
SRR5127858	Blastocystis sp. subtype 2	944160	21	317	100.00%	37.38%	98.91%
SRR5127860	Saccharomyces cerevisiae S288C	559292	20	45	10.99%	6.84%	99.67%

Table S3. Difference in EukDetect results for the 1000IBD and 500FG studies between pre- and post-rarefaction.

**Genus**	**Disease status**	**Abundance before rarefying**	**Abundance after rarefying**	**Difference in abundance**
Blastocystis	control	133	95	38
Blastocystis	IBD	20	14	6
Candida	control	1	1	0
Candida	IBD	6	2	4
Clavispora	IBD	1	1	0
Cyberlindnera	control	2	1	1
Cyberlindnera	IBD	1	0	1
Debaryomyces	control	4	1	3
Debaryomyces	IBD	4	3	1
Giardia	control	3	0	3
Hanseniaspora	control	1	0	1
Malassezia	control	2	0	2
Malassezia	IBD	1	1	0

Meyerozyma	IBD	1	0	1
Nakaseomyces	IBD	8	6	2
Penicillium	control	8	1	7
Penicillium	IBD	4	1	3
Pichia	control	3	1	2
Saccharomyces	control	46	13	33
Saccharomyces	IBD	64	31	33
Wickerhamomyces	IBD	1	1	0

Table S4. Results of EukDetect for the faecal microbiota transplant study data.

Sample ID	Taxa	Taxa ID	Observed markers	Read counts	Percent observed markers	Total marker coverage	Percent identity
SRR11599159	Pichia fermentans	53655	3	5	2.04%	8.77%	99.53%
SRR11599159	Candida tropicalis MYA-3404	294747	2	4	1.17%	6.34%	99.20%
SRR11599155	Saccharomyces cerevisiae S288C	559292	68	95	37.36%	7.76%	99.55%
SRR11599156	Saccharomyces cerevisiae S288C	559292	10	10	5.49%	4.81%	100.00%
SRR11599156	Penicillium nalgiovense	60175	5	6	2.42%	4.33%	100.00%
SRR11599156	Debaryomyces hansenii CBS767	284592	4	5	2.19%	7.90%	100.00%
SRR11599156	Penicillium	5073	3	4	0.56%	9.15%	100.00%
SRR11599153	Saccharomyces cerevisiae S288C	559292	5	5	2.75%	5.10%	100.00%
SRR11599154	Saccharomyces cerevisiae S288C	559292	14	17	7.69%	7.22%	99.73%
SRR11599150	Penicillium roqueforti FM164	1365484	4	4	2.01%	4.87%	100.00%
SRR11599152	Penicillium roqueforti FM164	1365484	3	4	1.51%	8.82%	100.00%

SRR11599149	Penicillium roqueforti FM164	1365484	3	4	1.51%	5.67%	100.00%
SRR11599143	Pichia kluyveri	36015	13	19	12.26%	6.89%	98.96%
SRR11599143	Torulaspora delbrueckii	4950	3	4	1.71%	6.25%	98.30%
SRR11599144	Clavispora lusitaniae ATCC 42720	306902	10	10	5.78%	3.22%	99.32%
SRR11599142	Blastocystis sp. subtype 2	944160	21	202	100.00%	43.40%	98.83%
SRR11599141	Saccharomyces cerevisiae S288C	559292	55	72	30.22%	7.18%	99.65%
SRR11599133	Blastocystis sp. subtype 4	944170	4	14	40.00%	20.04%	99.77%
SRR11599177	Blastocystis sp. ATCC 50177/Nand II	478820	11	32	55.00%	15.03%	99.21%
SRR11599129	Blastocystis sp. ATCC 50177/Nand II	478820	5	15	25.00%	13.24%	99.14%
SRR11599118	Blastocystis sp. ATCC 50177/Nand II	478820	10	14	50.00%	9.23%	99.03%
SRR11599096	Blastocystis sp. ATCC 50177/Nand II	478820	10	22	50.00%	12.12%	99.03%
SRR11599151	Blastocystis sp. ATCC 50177/Nand II	478820	10	14	50.00%	8.60%	99.36%
SRR11599140	Blastocystis sp. ATCC 50177/Nand II	478820	3	4	15.00%	8.52%	98.87%
SRR11599114	Penicillium roqueforti FM164	1365484	3	6	1.51%	4.66%	100.00%
SRR11599110	Candida albicans SC5314	237561	13	15	7.74%	5.70%	99.75%
SRR11599110	Saccharomyces cerevisiae S288C	559292	8	8	4.40%	9.10%	100.00%
SRR11599109	Saccharomyces cerevisiae S288C	559292	10	13	5.49%	5.44%	99.77%
SRR11599108	Pichia kudriavzevii	4909	5	5	3.16%	3.04%	100.00%
SRR11599106	Saccharomyces cerevisiae S288C	559292	5	5	2.75%	6.85%	100.00%
SRR11599106	Diutina catenulata	45537	3	5	1.99%	7.74%	99.54%
SRR11599162	Debaryomyces hansenii CBS767	284592	7	8	3.83%	7.69%	99.71%
SRR11599162	Penicillium roqueforti FM164	1365484	6	6	3.02%	4.57%	100.00%
SRR11599162	Saccharomyces cerevisiae S288C	559292	4	4	2.20%	6.86%	99.60%
SRR11599122	Saccharomyces cerevisiae S288C	559292	11	11	6.04%	4.93%	99.86%
SRR11599123	Saccharomyces cerevisiae S288C	559292	22	26	12.09%	5.62%	99.82%
SRR11599123	Pichia kudriavzevii	4909	4	4	2.53%	2.87%	99.81%
SRR11599117	Saccharomyces cerevisiae S288C	559292	10	12	5.49%	7.60%	99.55%

SRR11599116	Saccharomyces cerevisiae S288C	559292	49	69	26.92%	7.85%	99.61%
SRR11599121	Saccharomyces cerevisiae S288C	559292	19	20	10.44%	6.22%	99.66%
SRR11599120	Saccharomyces cerevisiae S288C	559292	8	8	4.40%	4.91%	99.90%
SRR11599097	Saccharomyces cerevisiae S288C	559292	14	15	7.69%	5.42%	99.90%
SRR11599097	Candida albicans SC5314	237561	4	4	2.38%	9.14%	100.00%
SRR11599098	Saccharomyces cerevisiae S288C	559292	37	48	20.33%	7.23%	99.75%
SRR11599098	Candida albicans SC5314	237561	5	5	2.98%	4.62%	99.55%
SRR11599098	Debaryomyces hansenii CBS767	284592	4	5	2.19%	7.85%	99.85%
SRR11599095	Saccharomyces cerevisiae S288C	559292	69	115	37.91%	8.89%	99.78%
SRR11599169	Blastocystis sp. ATCC 50177/Nand II	478820	15	74	75.00%	23.55%	99.18%
SRR11599078	Penicillium roqueforti FM164	1365484	4	4	2.01%	5.70%	100.00%
SRR11599075	Penicillium roqueforti FM164	1365484	7	7	3.52%	4.66%	100.00%
SRR11599073	Penicillium roqueforti FM164	1365484	5	5	2.51%	5.15%	100.00%
SRR11599072	Penicillium roqueforti FM164	1365484	33	38	16.58%	6.69%	100.00%
SRR11599076	Penicillium roqueforti FM164	1365484	4	4	2.01%	6.38%	100.00%
SRR11599069	Saccharomyces cerevisiae S288C	559292	20	26	10.99%	6.11%	99.77%
SRR11599069	Debaryomyces hansenii CBS767	284592	4	4	2.19%	5.74%	100.00%
SRR11599066	Debaryomyces hansenii CBS767	284592	92	181	50.27%	10.84%	99.41%
SRR11599066	Kluyveromyces lactis	28985	7	9	4.29%	4.15%	99.91%
SRR11599066	Kluyveromyces marxianus DMKU3-1042	1003335	4	5	2.44%	8.19%	99.85%
